# Titanium Alloy Ti-6Al-4V Electrochemical Dissolution Behavior in NaNO_3_ and NaCl Solutions at Low Current Density

**DOI:** 10.3390/ma17205026

**Published:** 2024-10-14

**Authors:** Shen Niu, Changyang Yu, Pingmei Ming, Siru Wang, Ge Qin, Xinchao Li, Huan Liu

**Affiliations:** School of Mechanical and Power Engineering, Henan Polytechnic University, Jiaozuo 454003, China; 19513206500@163.com (C.Y.); mingpingmei@163.com (P.M.); 18855033057@163.com (S.W.); qinge@hpu.edu.cn (G.Q.); hpulixinchao@163.com (X.L.); liuhuan@hpu.edu.cn (H.L.)

**Keywords:** jet electrochemical micromilling, stray corrosion, low current density, Ti-6Al-4V, passive film

## Abstract

Jet electrochemical micromilling (JEMM) exhibits significant potential for high-efficiency and high-quality machining of titanium alloy microstructures. However, during the JEMM process, the machined surface of the workpiece inevitably experiences stray current attacks at low current levels. Due to the formation of a dense passive film on the surface of the titanium alloy under electrochemical action, stray corrosion occurs on the machined surface. Hence, the electrochemical dissolution behavior of titanium alloys at low current densities directly impacts both machining efficiency and quality. This study first analyzed the effects of electrolyte composition and current density on the transpassive potential, breakdown of the passive film, current efficiency, and the dissolved surface on Ti-6Al-4V. The transpassive potential and electrochemical impedance of Ti-6Al-4V were found to be lower in NaCl solution than in NaNO_3_ solution. In addition, lower current densities enabled higher current efficiency and resulted in a more uniform and flat dissolution surface. Subsequent experiments used these two solutions for JEMM of complex-shaped microstructures on Ti-6Al-4V. The findings demonstrated that, compared to the NaNO_3_ solution, the use of NaCl solution increases the material removal rate by approximately 30%, enhances the aspect ratio by about 26%, and reduces surface roughness by roughly 58%. This indicates that employing NaCl solution can lead to superior machining efficiency and quality.

## 1. Introduction

Titanium alloys are widely used in medical devices, aerospace applications, and other fields due to their low density, high specific strength, excellent biocompatibility, and outstanding corrosion and heat resistance [1,2]. The fabrication of micro-miniature components and critical parts with intricate microstructures has become increasingly vital for advancing the high-end equipment industry [3,4]. However, titanium alloys are considered difficult-to-machine materials because of their low elastic modulus, poor thermal conductivity, high toughness, and strong chemical affinity, significantly restricting their application in metal microstructures [5,6].

Jet electrochemical micromilling (JEMM) is a micro-electrochemical milling technique that employs a metal nozzle as a tool cathode, combined with multi-axis CNC milling motion, to remove metallic materials based on the principle of electrochemical anodic dissolution [7]. This technology transcends the mechanical properties of materials and is characterized by the absence of residual stress, recast layers, and microcracks, presenting significant advantages in machining metal micro-components. However, the strong passivation tendency of titanium leads to the formation of a dense passive film on the surface of titanium alloys under electrochemical conditions, which impedes electrolytic machining and affects both efficiency and quality [8]. Therefore, the electrolytic machining of macro-scale titanium alloy parts often employs NaCl solution known for its strong activation capability. Xu et al. [9] reported that NaCl solution produces a smoother and more uniform dissolved surface compared to NaNO_3_ solution. The Ti60 integral blade disk was manufactured using NaCl solution, featuring a surface roughness *R*a of 0.6 μm and machining accuracy of 0.05 mm. He et al. [10] used a 10% NaCl solution for electrolytic machining of Ti-6Al-4V and noted that when the current density increases from 16 to 112.5 A/cm^2^, the surface roughness *R*a of the titanium alloy decreases from 1.3 to 0.4 μm, significantly enhancing the surface quality. Weinmann M et al. [11] demonstrated that as the NaCl solution concentration increased, so did the current density and the electrochemical dissolution rate for titanium alloys Ti90Al6V4 and Ti60Al40, achieving higher machining efficiency.

Traditional electrochemical machining typically utilizes the end-face feed method for the tool cathode, with the final surface shape of the workpiece being formed by the end-face gap. In contrast, the final surface shape in electrochemical milling is mainly shaped by the side gap, leading to stray current corrosion on the workpiece’s machined surface due to the movement of the tool cathode [7]. Compared to NaCl solution, NaNO_3_ solution induces a strong passivation effect, enhancing the transpassive voltage of the workpiece anode, reducing stray current corrosion on both unprocessed and processed surfaces, and improving the precision and quality of electrochemical milling [12,13,14]. In recent years, macro-electrochemical milling of titanium alloys has frequently utilized NaNO_3_ solutions for improved processing localization. Liu et al. [15] achieved high-speed and uniform dissolution of titanium alloy TB6 in a 20% NaNO_3_ solution at a current density of 140 A/cm^2^, creating grooves and flat surfaces with favorable surface quality. Wang et al. [16] utilized a 20% NaNO_3_ solution for macro-electrochemical milling of Ti-6Al-4V and found that when the current density exceeded 70 A/cm^2^, the time required to break the surface passive film stabilized at 0.58 s. They enhanced the processing localization and surface quality of the grooves by optimizing the cathode feed rate. Liu et al. [17] used a 10% NaNO_3_ solution and a cutting depth of 3 mm to process Ti-6Al-4V by cut-in electrochemical milling. They achieved a material removal rate of 40.62 mm^3^/min, with a maximum groove depth of 3.81 mm and a surface roughness *R*a of 4.853 μm by increasing voltage and feed speed.

Currently, the predominant materials processed by JEMM for metal components are stainless steel and nickel-based superalloys [18,19,20]. This is because, compared with the macro electrochemical milling process, the conductive area of the metal nozzle in JEMM is smaller, resulting in a low processing current on the machining surface of the workpiece, which leads to a reduced stray current on the machined surface. When utilizing NaNO_3_ solution, the passive film on the surface of titanium alloys forms more rapidly, becomes denser, and thickens, requiring a higher transpassive potential to break the passive film. Therefore, at low stray currents, the passive film on the machined surface of titanium alloy is challenging to remove swiftly and uniformly, which inevitably affects the machining quality of the final surface in JEMM. Clearly, the material properties of titanium alloys significantly restrict the application of JEMM, making it urgent to conduct relevant theoretical and experimental research.

This study investigates the electrochemical dissolution characteristics of titanium alloy Ti-6Al-4V in NaNO_3_ and NaCl solutions through polarization curves, electrochemical impedance spectroscopy, and current efficiency curve tests. It focuses on analyzing the impact mechanisms of electrolyte composition and current density on the transpassive potential, breakdown of the passive film, current efficiency, and the dissolved surface of Ti-6Al-4V. In addition, this research compares the material removal rate, aspect ratio, and surface roughness of jet electrochemical micromilling of complex-shaped Ti-6Al-4V microstructures in these two solutions. The results show that NaCl solutions can achieve higher processing efficiency and quality.

## 2. Experimental Details

### 2.1. Material Preparation

The samples employed in this experiment consist of the titanium alloy Ti-6Al-4V, which is primarily used in the biomedical, aerospace, and precision instrument industries. This alloy, classified as the “α + β” type, ranks among the most extensively utilized titanium alloys; its chemical composition is detailed in Table 1. The material was sectioned into samples measuring 10 × 10 × 10 mm, 5 × 5 × 10 mm, and 30 × 20 × 5 mm using electrical discharge wire cutting. Before the experiments, the working surfaces of the samples were ground with silicon carbide sandpaper (from 2000 mesh to 7000 mesh) and then polished with a polishing cloth until a mirror-like surface was achieved. The samples were then cleaned with anhydrous ethanol using ultrasonic cleaning equipment for 30 min to ensure surface smoothness and cleanliness.

### 2.2. Electrochemical Testing

**Figure 1:**
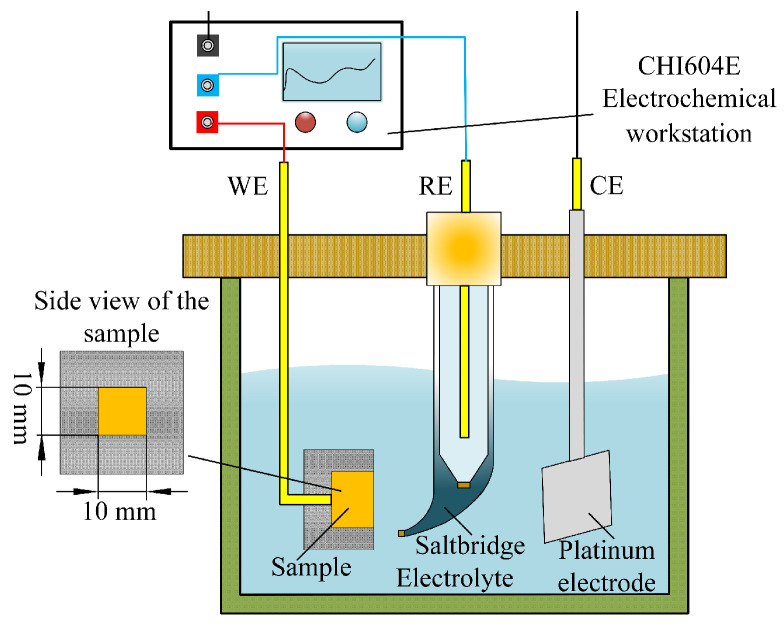
Schematic view of the device used to measure polarization curves and EIS.

In electrochemical machining, electrochemical testing is generally used to study the electrochemical behavior of materials in specific electrolytes and their dissolution characteristics [21]. In this study, an electrochemical workstation (CHI604E, CH Instruments, Shanghai, China) was used to perform electrochemical testing on Ti-6Al-4V in various solutions (including polarization curve measurements and electrochemical impedance spectroscopy). Figure 1 depicts the test setup. A platinum electrode and a saturated calomel electrode (SCE) were utilized as the auxiliary electrode (CE) and reference electrode (RE). The Ti-6Al-4V sample, measuring 10 × 10 × 10 mm, was the working electrode (WE). The working electrode was positioned in a specific fixture, exposing an area of 1 cm^2^ to the solution. The solutions used in the experiment were 10 wt% NaNO_3_ and NaCl, prepared by mixing solid NaNO_3_ and NaCl salts (Tianjin Oubokai Chemical Co., Ltd., Binhai New Area, Tianjin, China) with distilled water, and maintained at 25 °C.

Before the polarization curve and electrochemical impedance spectroscopy (EIS) tests, the specimens, secured in the fixture, were immersed in NaNO_3_ and NaCl solutions for three hours under open circuit potential to stabilize their potential values. The Tafel polarization test was performed at a scan rate of 1 mV/s, with a scan potential range from −650 mV_SCE_ to 100 mV_SCE_. The potentiodynamic polarization test was conducted at a scan rate of 10 mV/s, with a scan potential range from −2 V_SCE_ to 8 V_SCE_. After completing the potentiodynamic polarization curve test, a scanning electron microscope (SEM) (Merlin Compact, Carl Zeiss NTS GmbH, Oberkochen, Germany) was used to examine the surface morphology of the specimen. Once the open circuit potential stabilized, electrochemical impedance spectroscopy (EIS) tests were performed. The frequency range for EIS was 100 kHz to 10 mHz, with a disturbance amplitude of 5 mV. EIS data were analyzed using ZView software (Version 3.3). To ensure the reliability of the experimental data, each set of experiments was repeated three times.

### 2.3. Current Efficiency Measurement

**Figure 2:**
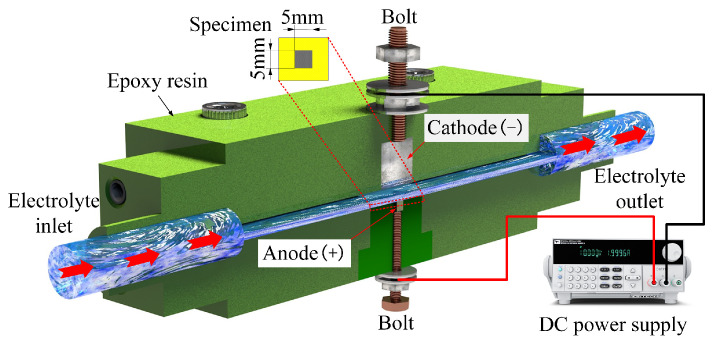
Schematic diagram of current efficiency measurement device.

Current efficiency indicates the ratio of the actual dissolved mass of the anode metal during electrochemical dissolution to the theoretically calculated mass. It is widely employed to gauge the removal efficiency of the specimen material at varying current densities [22]. Figure 2 shows the current efficiency test apparatus, with the specimen dimensions at 5 × 5 × 10 mm. During the experiment, the specimen was placed in an epoxy resin fixture slot, with only one surface (5 × 5 mm) facing the cathode exposed to the solution flow path, while the other five surfaces were encapsulated and sealed with epoxy resin. The test used 10 wt% NaNO_3_ and NaCl solutions, with the solution supply pressure maintained at 0.4 MPa. Current efficiency is primarily influenced by current density; therefore, it was evaluated at various current densities. A constant current method was utilized to maintain a steady current density. Before and after each test, the specimens were cleaned, dried, and weighed using a high-precision electronic balance (SQP, SARTORIUS, Göttingen, Germany) to determine the mass removal ∆*m* of the specimen material during the test. The anodic current efficiency was calculated using Equation (1) [22].

*η* = ∆*m*/(*ρ*_a_*Itω*) (1)

where *η* is the anodic current efficiency; *I* is the processing current value (A); *t* is the processing time (s); *ρ*_a_ is the density of the anode metal (g/cm^3^); *ω* is the electrochemical volume equivalent (cm^3^/A·s).

Ti-6Al-4V is composed of various metallic elements, and its electrochemical volume equivalent *ω* is calculated using Equation (2).

*ω* = 1/(*ρ*_a_*F*(*n*_1_*a*_1_/*A*_1_ + *n*_2_*a*_2_/*A*_2_ + ⋯ + *n*_j_*a*_j_/*A*_j_)) (2)

where *F* is the Faraday constant, *A*_1_, *A*_2_, …, *A*_j_ are the relative atomic masses of different elements; *n*_1_, *n*_2_, …, *n*_j_ are the valences of the atoms; *a*_1_, *a*_2_, …, *a*_j_ are the percentage contents of the elements, and *ρ*_a_ is 4.5 g/cm^3^. The electrochemical volume equivalent *ω* of Ti-6Al-4V is calculated to be 5.61 × 10^−4^ cm^3^/(A·min) by substituting the mass percentages of the elements listed in Table 1 into Equation (2).

After the recent efficiency tests, the surface morphology and chemical composition of Ti-6Al-4V under varying current densities in NaNO_3_ and NaCl solutions were analyzed using Scanning Electron Microscope (SEM) and Energy Dispersive X-ray Spectroscopy (EDS). The EDS data was collected under high vacuum conditions with an accelerating voltage of 8 kV and a collection time of 60 s.

## 3. Results and Discussion

### 3.1. Polarization Curve Test

**Figure 3:**
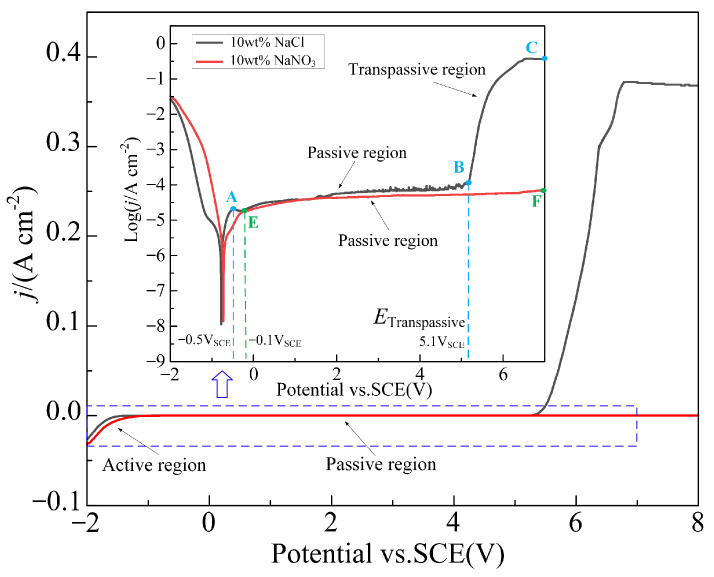
Potentiodynamic polarization curves of Ti-6Al-4V in NaNO_3_ and NaCl solutions.

**Figure 4:**
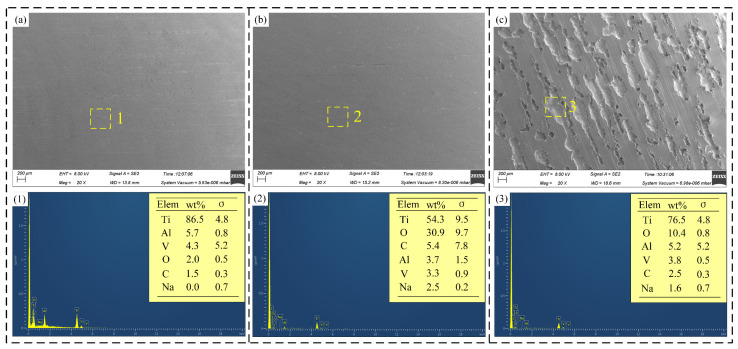
The figure shows the surface morphology and chemical composition of Ti-6Al-4V before polarization and after polarization in different solutions: (**a**) the surface before the polarization experiment; (**b**) the surface after polarization in NaNO_3_ solution; (**c**) the surface after polarization in NaCl solution; (**1**) chemical element composition of region 1; (**2**) chemical element composition of region 2; (**3**) chemical element composition of region 3.

Figure 3 illustrates the potentiodynamic polarization curves of Ti-6Al-4V in NaNO_3_ and NaCl solutions, while Figure 4 presents the surface morphology and chemical composition of Ti-6Al-4V before polarization and after polarization in different solutions. Within the measured range of polarization potential, Ti-6Al-4V exhibited activation-passivation electrochemical behavior in the NaNO_3_ solution, whereas in the NaCl solution, it showed distinct activation-passivation-transpassivation electrochemical behavior. When the polarization potential reaches approximately −0.1 V_SCE_ (NaNO_3_ solution) and −0.5 V_SCE_ (NaCl solution), Ti-6Al-4V enters the passive region, where the current density does not significantly change with increasing polarization potential [23]. Due to the strong oxidizing nature of NO_3_^−^ ions in the NaNO_3_ solution, the passive film formed on the surface of Ti-6Al-4V becomes denser and more stable, significantly increasing the difficulty of passive film breakdown. However, in the NaCl solution, when the polarization potential increases to around 5.1 V_SCE_, Ti-6Al-4V enters the transpassive region (B-C), where the passive film is breached and fractured, causing the current density to rise rapidly, and the substrate material begins to dissolve swiftly. As shown in Figure 4, compared to the surface before polarization, the surface morphology of Ti-6Al-4V after polarization in the NaNO_3_ solution shows no significant changes, with a marked increase in oxygen content and a significant decrease in titanium content, indicating the formation of a passive film on the surface. In contrast, after polarization in the NaCl solution, the Ti-6Al-4V surface exhibits numerous localized dissolution areas, where the oxygen content is noticeably lower compared to that in the NaNO_3_ solution, and the titanium content is significantly higher. This indicates that the passive film in localized areas of the Ti-6Al-4V surface has fractured, exposing the substrate material, which has undergone rapid corrosion and dissolution. This is because, compared to NO_3_^−^ ions, Cl^−^ ions in the NaCl solution can penetrate the weaker areas of the passive film, leading to dissolution at the interface between the passive film and the substrate material, thereby causing the passive film to fracture.

**Figure 5:**
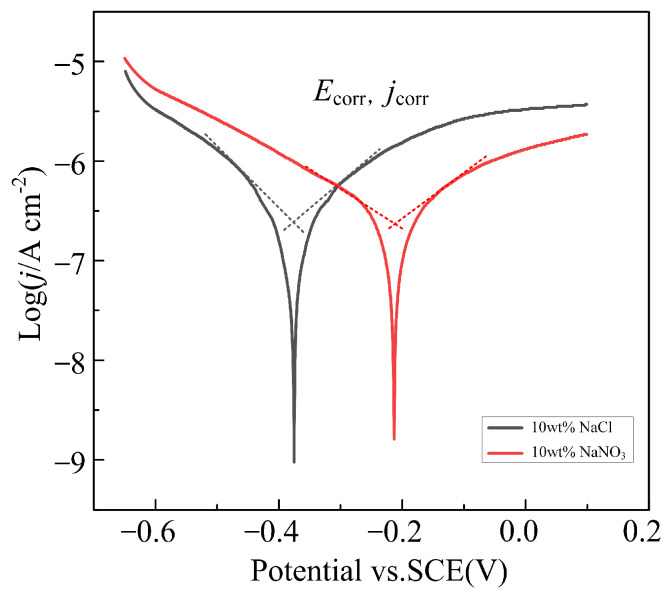
Tafel polarization curves of Ti-6Al-4V in NaNO_3_ and NaCl solutions.

Figure 5 illustrates the Tafel polarization curves of Ti-6Al-4V in NaNO_3_ and NaCl solutions. Table 2 presents the corrosion potential (*E*_corr_) and corrosion current density (*j*_corr_) calculated using the extrapolation method from the polarization curves. Generally, the more positive the corrosion potential, the better the corrosion resistance of the material; according to Faraday’s law, the corrosion current density is positively correlated with the corrosion rate [24,25]. The results show that the corrosion potential of Ti-6Al-4V in NaCl solution (−375.63 ± 6.55 mV_SCE_) is significantly lower than that in NaNO_3_ solution (−212.42 ± 2.93 mV_SCE_), indicating that its corrosion resistance in NaCl solution is poorer, making it more susceptible to electrochemical dissolution. Additionally, the corrosion current density of Ti-6Al-4V in NaCl solution (242.49 ± 5.82 nA/cm^2^) is higher than that in NaNO_3_ solution (181.97 ± 4.31 nA/cm^2^), suggesting a higher corrosion rate in the NaCl solution. Therefore, when performing jet electrochemical micromachining of Ti-6Al-4V in NaCl solution, the transpassive voltage of the passive film is lower, making the passive film more prone to rupture, and the material achieves a higher dissolution rate.

### 3.2. Electrochemical Impedance Test

**Figure 6:**
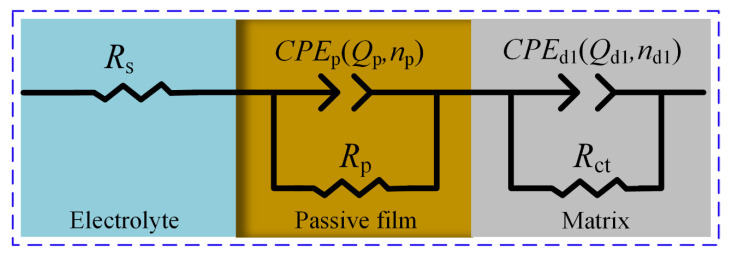
The EEC model used for fitting the EIS data of Ti-6Al-4V in NaNO_3_ and NaCl solutions.

EIS was utilized to explore the passive film’s electrochemical properties on the sample surface. EIS measurements were conducted at open circuit potential. The experimental results showed that the OCP values for Ti-6Al-4V in NaNO₃ and NaCl solutions were approximately 0.2 V_SCE_ and 0.035 V_SCE_, respectively. Combined with the polarization curve tests in Section 3.1, these potential values indicate that the potential of Ti-6Al-4V was within the passivation region, suggesting that a passive film had formed on its surface. The EIS data, as illustrated in Figure 6, were modeled using an equivalent electrical circuit (EEC) that incorporates the resistance of the electrolyte (*R*_s_), the passive film resistance (*R*_p_), and the charge transfer resistance (*R*_ct_). Constant phase elements (CPE1 and CPE2) were included, with their impedance calculated by Equation (3) [26]:

*Z*_CPE_ = [*Q*(*iω*)^n^]^−1^ (3)

where *Q* is the CPE constant, *i* denotes the imaginary unit (*i*^2^ = −1); *ω* is the angular frequency (rad/s); *n* is the CPE exponent (with *n* = 1 for an ideal electrode surface). *Q*_p_ indicates the passive film capacitance, while *Q*_d1_ denotes the double-layer capacitance, *n*_p_ and *n*_d1_ are the exponents of capacitance, used to describe the deviation between actual capacitance and ideal capacitance.

**Figure 7:**
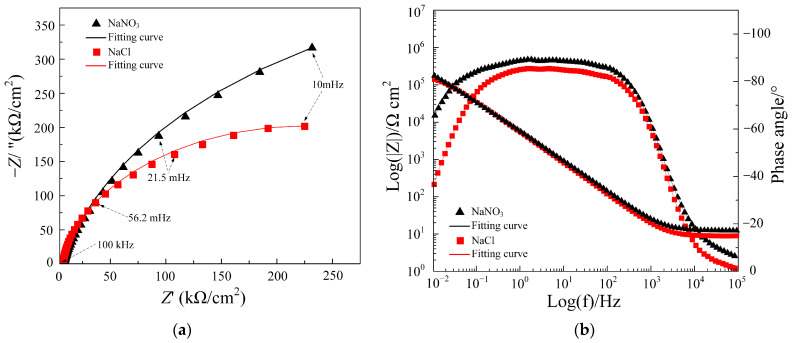
EIS results of Ti-6Al-4V in NaNO_3_ and NaCl solutions: (**a**) Nyquist plot; (**b**) Bode plot.

Figure 7 indicates that Nyquist and Bode plots represent the EIS data for Ti-6Al-4V in NaNO_3_ and NaCl solutions. Figure 7a reveals that the Nyquist curves of Ti-6Al-4V in both solutions exhibit a capacitive response across nearly the entire frequency range. The capacitive semicircle’s diameter correlates with the passive film’s charge transfer resistance. A larger semicircle diameter indicates that the formed passive film has better corrosion resistance. This result is consistent with the relationship between passive film impedance and its corrosion resistance as described in references [14,27]. The Nyquist curve diameter in NaCl solution is considerably smaller than in NaNO_3_ solution, indicating that the passive film on Ti-6Al-4V in NaCl solution has relatively poor corrosion resistance and is more susceptible to breakdown and electrochemical dissolution. In addition, the Bode plot in Figure 7b shows that the impedance modulus and the absolute value of the phase angle at low frequencies for Ti-6Al-4V in NaCl solution are both lower than those in NaNO₃ solution. The lower phase angle indicates that the passive film formed on Ti-6Al-4V in NaCl solution is less compact. This suggests that the passive film is more loose, leading to reduced impedance. Concurrently, the lower impedance modulus signifies that the passive film has lower resistance, which facilitates faster ion exchange between the solution and the substrate, resulting in an increased rate of electrochemical reactions. Therefore, Ti-6Al-4V exhibits a higher electrochemical reaction rate in NaCl solution.

Table 3 lists the data derived from fitting the equivalent circuit. The Χ^2^ values, on the order of 10^−4^, indicate an excellent fit between the measured data and the model. The *R*_ct_ value indicates the difficulty of charge transfer at the electrode interface with the solution. In the NaCl solution, the *R*_ct_ value for Ti-6Al-4V is markedly lower than in the NaNO_3_ solution, showing that the charge transfer resistance is lower and electrochemical activity is higher in the NaCl solution. This conclusion is consistent with the findings in reference [11], where titanium alloys exhibited high electrochemical activity in chloride solutions. In addition, *Q*_p_ represents the capacitive behavior of the passive film, a higher *Q*_p_ value indicates a higher capacitance of the passive film, suggesting that the passive film is denser and more resistant to corrosion. *R*_p_ represents the resistance of the passive film, and a smaller *R*_p_ value indicates that the passive film is relatively weaker. The relatively lower *Q*_p_ and *R*_p_ values for Ti-6Al-4V in NaCl solution suggest that the passive film formed in this environment is relatively loose, making it more susceptible to ion penetration and electrochemical dissolution. Using NaCl solution for jet electrochemical micromilling of Ti-6Al-4V thus forms a passive film with lower impedance on the anode surface, promoting higher electrochemical reaction rates and more uniform material dissolution.

### 3.3. Current Efficiency Measurement

**Figure 8:**
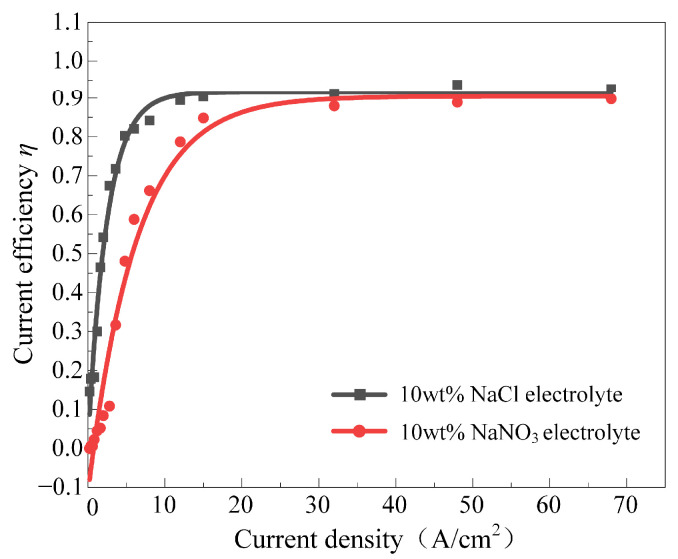
The relationship between current efficiency and current density of Ti-6Al-4V in NaNO_3_ and NaCl solutions.

Figure 8 illustrates the current density–current efficiency curves for Ti-6Al-4V in 10 wt% NaNO_3_ and NaCl solutions. In both solutions, the current efficiency of Ti-6Al-4V first increases and then stabilizes with the increase in current density. During the increasing phase, the current efficiency rapidly rises with the increase in current density. In the stabilization phase, the current efficiency of Ti-6Al-4V remains unchanged with further increases in current density, and the efficiency values of the two solutions are close.

However, during the increasing phase, the current efficiency of Ti-6Al-4V in the NaCl solution rises at a significantly higher rate than the NaNO_3_ solution and enters the stabilization phase of current efficiency earlier. This difference is attributable to the presence of a large amount of oxidative NO_3_^−^ ions in the NaNO_3_ solution, which contributes to a denser and more stable passive film on the surface of Ti-6Al-4V during electrochemical machining, increasing the difficulty of anodic dissolution. In contrast, the Cl^−^ ions in NaCl solution can easily penetrate the passive film, disrupting it from within and accelerating the substrate material’s dissolution. In addition, at high current density, a high electrode voltage forms between the cathode and the anode, which can quickly break down the passive film on the sample’s surface, exposing the substrate material to the solution, achieving high-speed material dissolution and obtaining high current efficiency. Hence, Ti-6Al-4V maintains stable current efficiency with increasing current density in both solutions, eventually reaching about 90%.

**Figure 9:**
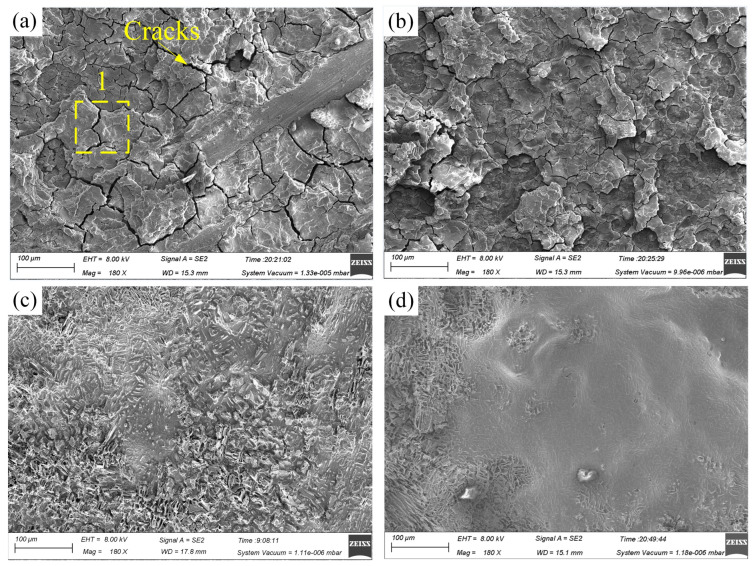
Surface morphologies of Ti-6Al-4V in NaNO_3_ solution at different current densities and processing times: (**a**) 2 A·cm^−2^ and 240s, (**b**) 3.6 A·cm^−2^ and 240 s, (**c**) 15 A·cm^−2^ and 120 s, and (**d**) 48 A·cm^−2^ and 40 s. (The yellow box indicates the analysis area, and the number 1 marks a specific location).

**Figure 10:**
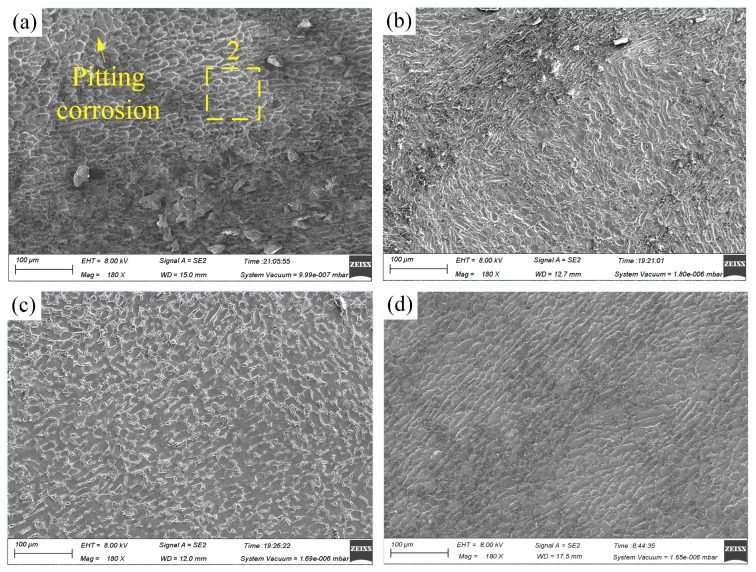
Surface morphologies of Ti-6Al-4V in NaCl solution at different current densities and processing times: (**a**) 2 A·cm^−2^ and 240 s, (**b**) 3.6 A·cm^−2^ and 240 s, (**c**) 15 A·cm^−2^ and 120 s, and (**d**) 48 A·cm^−2^ and 40 s. (The yellow box indicates the analysis area, and the number 2 marks a specific location).

**Figure 11:**
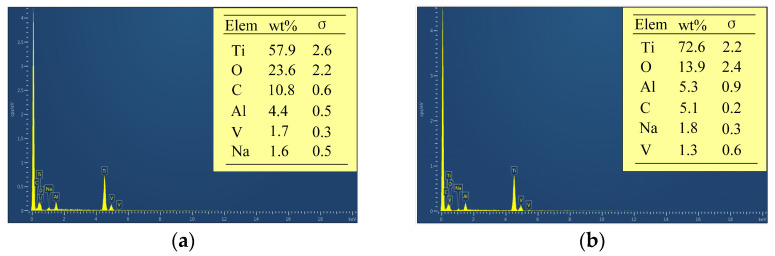
EDS analysis results of different regions on the dissolved surface of Ti-6Al-4V: (**a**) Region 1; (**b**) Region 2.

Figure 9 illustrates the surface morphology of Ti-6Al-4V in NaNO_3_ solution across various current densities and processing times. Figure 10 depicts the surface morphology of Ti-6Al-4V in NaCl solution under varying current densities and processing times. Figure 11 shows the EDS analysis results of different regions on the dissolved surface of Ti-6Al-4V. At a current density of 2 A·cm^−2^, high-magnification images reveal numerous cracks on the dissolved surface. EDS analysis of region 1, as depicted in Figure 11a, shows an oxygen content of approximately 23.6 wt%, indicating that these cracks result from the rupture of the passive film on the dissolved surface. As the current density increases to 3.6 A·cm^−2^, the passive film in certain areas begins to flake off. At a current density of 15 A·cm^−2^, the passive film substantially disappears, exposing the base material. However, the surface left behind is very rough, with uneven dissolution of the material. An increase in current density to 48 A·cm^−2^ expands the base material’s exposed area, improving the dissolved surface’s uniformity. This demonstrates that increasing the current density enhances the uniformity of the electrochemical dissolution, improving surface quality.

Compared to the NaNO_3_ solution, significant differences in the dissolved surface morphology are evident. The dissolved surface displays numerous pitting areas at a current density of 2 A·cm^−2^. EDS analysis of the chemical element composition in region 2, as shown in Figure 11b, found that the O element content accounts for only about 13.9 wt%, indicating that the passive film had broken and the base material was exposed. With an increase in current density to 3.6 A/cm^−2^, the uniformity of the dissolved surface improves, and the exposed area of the base material enlarges further. At a current density of 15 A·cm^−2^, the electrochemical dissolution reaction intensifies, resulting in increased pitting areas and an expanded spatial distribution. As the current density rises to 48 A·cm^−2^, the pitting areas become closely interconnected and overlap, yielding a smooth dissolved surface with superior surface quality.

At current densities of 2 A·cm^−2^ and 3.6 A·cm^−2^, the passive film on Ti-6Al-4V in NaCl solution has already fractured, whereas in NaNO_3_ solution, the passive film exhibits only cracks and partial flaking. At a current density of 15 A·cm^−2^, the dissolved surface obtained in the NaCl solution shows relatively superior uniformity. This phenomenon can be attributed to the activation effect of Cl^−^ ions on the passive film of Ti-6Al-4V in the NaCl solution, consistent with the findings in Section 3.1 and Section 3.2. These observations indicate that at lower current densities, the passive film on Ti-6Al-4V in NaCl solution is more prone to rupture, leading to a smoother and more uniform dissolved surface.

During the jet electrochemical micromilling process, the metal nozzle faces the machining surface, enabling the surface to achieve high current density. At high current densities, the passive film formed on the surface of Ti-6Al-4V quickly breaks down, allowing for efficient and rapid material dissolution. However, in the metal nozzle scanning process, the machined surface of the workpiece is mainly corroded by low stray current. Due to the small conductive area of the metal nozzle and the low processing current, the stray current affecting the machined surface of the workpiece remains relatively low. Hence, during jet electrochemical micromilling of Ti-6Al-4V, the machining surface can achieve efficient and rapid dissolution in both solutions. However, for the machined surface, Cl^−^ ions in NaCl solution can easily penetrate the passive film, effectively breaking it even at low current densities, leading to more uniform material dissolution. Therefore, employing NaCl solution for jet electrochemical micromilling of Ti-6Al-4V results in a smoother and more uniform processed surface.

### 3.4. Jet Electrochemical Micromilling Experiment

**Figure 12:**
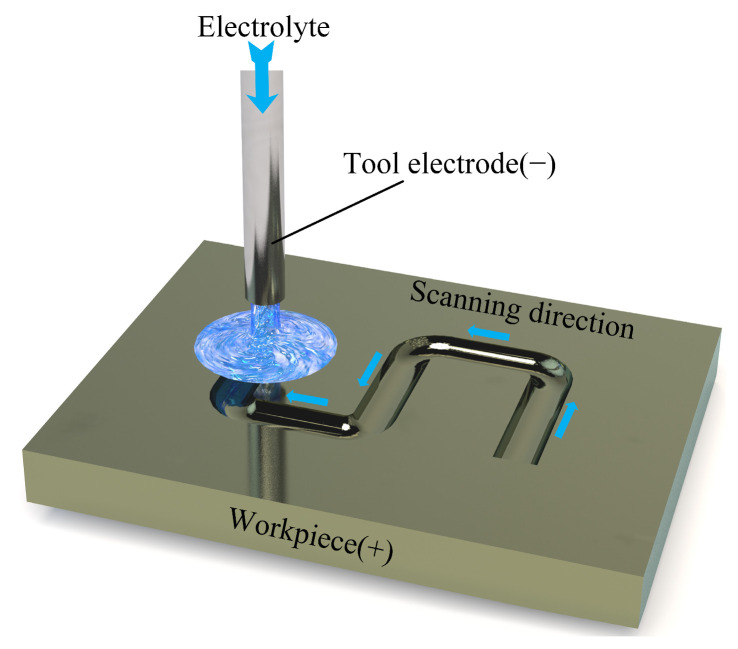
Schematic diagram of the principle of jet electrochemical micromilling.

This section uses NaNO_3_ and NaCl solutions with a temperature of 25 °C and a mass fraction of 10 wt% to conduct experimental research on the microstructure of Ti-6Al-4V through jet electrochemical micromilling. The metal nozzle used in the test has an inner diameter of 0.3 mm and an outer diameter of 0.6 mm. Figure 12 demonstrates that during the jet electrochemical micromilling process, the electrolyte is continuously supplied through the metal nozzle, impacting the surface of the workpiece. In this setup, the metal nozzle functions as the cathode and the workpiece as the anode, with the metal nozzle’s trajectory precisely controlled by a numerical control (NC) program. A conductive circuit is formed during the machining process, and an electrochemical anodic dissolution reaction removes the material of the workpiece. According to the machining test parameters shown in Table 4, by setting the scanning trajectory, the surface of the Ti-6Al-4V sample measuring 30 × 20 × 5 mm was machined to create an “S”-shaped microstructure. Then, a laser confocal microscope (OLS5100, Olympus, Japan) was employed to conduct a three-dimensional morphology scan of the microstructure, assessing its groove depth, groove width, and surface roughness (*R*a). In addition, a high-precision electronic balance (SQP, SARTORIUS, Germany) was utilized to measure the weight of the workpiece before and after the experiment, calculating the mass difference (∆*m*) and recording the processing time (*t*). The material removal rate (*MRR*) of Ti-6Al-4V was then calculated using Equation (4).

*MRR* = ∆*m*/(*ρ*_a_*t*) (4)

**Figure 13:**
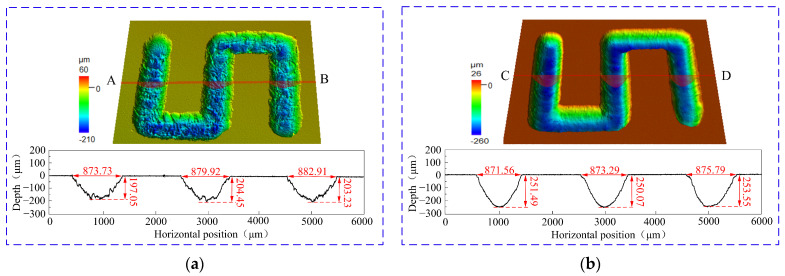
3D morphology of the “S”-shaped microstructures of jet electrochemical micromilling under different solutions: (**a**) NaNO_3_ solution (**b**) NaCl solution.

**Figure 14:**
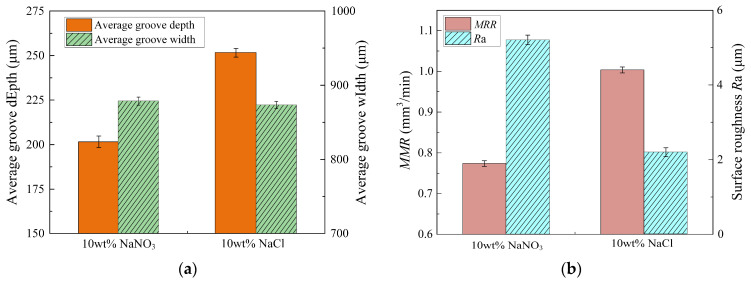
Machining performance of the “S”-shaped microstructure machined via jet electrochemical micromilling using NaNO_3_ and NaCl solutions: (**a**) Average width and depth; (**b**) *MRR* and *R*a.

Figure 13 presents the “S”-shaped microstructures fabricated using different electrolytic solutions in the jet electrochemical micromilling process. Figure 13a illustrates the microstructure fabricated with NaNO_3_ solution, including cross-section A-B, while Figure 13b depicts the microstructure fabricated with NaCl solution, including cross-section C-D. The average depths and widths of the grooves in these cross-sections were measured, with results in Figure 14a. Figure 14b illustrates the *MRR* and *R*a of Ti-6Al-4V when subjected to jet electrochemical micromilling in various solutions. When using NaNO_3_ solution for jet electrochemical micromilling, the measured aspect ratio of the microstructure was 0.23 (standard deviation of 0.012). The *R*a was assessed at three arbitrary locations on the microstructure’s bottom surface, yielding an average value of 5.194 μm (standard deviation of 0.17 μm). In contrast, when using NaCl solution for jet electrochemical micromilling, the aspect ratio of the microstructure was found to be 0.29 (standard deviation of 0.017), with *R*a measured by the same method yielding 2.18 μm (standard deviation of 0.12 μm). In addition, Figure 14b indicates that the material removal rate of Ti-6Al-4V is 0.7711 mm^3^/min (standard deviation of 0.013) in NaNO_3_ solution and 1.0023 mm^3^/min (standard deviation of 0.018) in NaCl solution.

Experimental results indicate that compared to NaNO_3_ solution, using NaCl solution for jet electrochemical micromilling of Ti-6Al-4V enhances the material removal rate by approximately 30%, increases the aspect ratio by approximately 26%, and reduces surface roughness by approximately 58%. This attributes to Cl^−^ ions can effortlessly penetrate the passive film on the Ti-6Al-4V surface, facilitating rapid and uniform removal of the passive film on the machined surface of the titanium alloy, even under low stray currents. The processed products are quickly removed by a high-speed jet, thus achieving a higher-quality machined profile. This finding aligns with the research predictions presented in Section 3.3. Accordingly, employing NaCl solution for jet electrochemical micromilling of Ti-6Al-4V achieves higher processing efficiency, improved accuracy, and superior surface quality.

## 4. Conclusions

This study explores the electrochemical dissolution characteristics of Ti-6Al-4V in NaNO_3_ and NaCl solutions, with a focus on analyzing the impact mechanisms of electrolyte composition and current density on the transpassive potential, breakdown of the passive film, current efficiency, and the dissolved surface of Ti-6Al-4V. Experiments utilizing jet electrochemical micromilling with these two solutions were then conducted to fabricate complex-shaped microstructures of Ti-6Al-4V. The research yielded the following conclusions:

1. The polarization curve and EIS results demonstrate that the passive film’s transpassive potential and electrochemical impedance on Ti-6Al-4V in the NaCl solution are significantly lower than those observed in the NaNO_3_ solution. This indicates that the passive film in NaCl solution is more prone to breakdown, leading to an increased electrochemical dissolution rate.
2. The current efficiency tests reveal that at low current densities, Ti-6Al-4V exhibits higher current efficiency and a smoother, more uniform dissolution surface in NaCl solution than in NaNO_3_ solution. These findings demonstrate that using NaCl solution in jet electrochemical micromilling of Ti-6Al-4V promotes the rapid and uniform breakdown of the passive film on the machined surface, enhancing surface quality.
3. Results from jet electrochemical micromilling experiments indicate that, compared to NaNO_3_ solution, employing NaCl solution increases the *MRR* of the microstructure by approximately 30%, enhances the aspect ratio by about 26%, and reduces *R*a by roughly 58%. These results show that Ti-6Al-4V can be effectively machined using jet electrochemical micromilling with NaCl solution, achieving higher machining efficiency, precision, and superior surface quality.

## Figures and Tables

**Table 1:** Chemical composition of titanium alloy Ti-6Al-4V (wt%).

Element	Ti	V	Fe	Al	H	O	N	C
Mass fraction (%)	89.33	4	0.3	6	0.015	0.2	0.05	0.1

**Table 2:** Corrosion parameters evaluated from polarization curves of Ti-6Al-4V.

Sample	*E*_corr_ (mV_SCE_)	*j*_corr_ (nA cm^−2^)
NaNO_3_	−212.42 ± 2.93	181.97 ± 4.31
NaCl	−375.63 ± 6.55	242.49 ± 5.82

**Table 3:** Fitting parameters of Ti-6Al-4V EEC model in different solutions.

Parameters	NaNO_3_	NaCl
*R*_s_ (Ω/cm^2^)	7.98	5.49
*Q*_p_ (s^n^_p_ Ω^−1^ cm^−2^)	1.91 × 10^−4^	1.51 × 10^−4^
*n*_p_	0.89	0.92
*R*_p_ (Ω/cm^2^)	4.64 × 10^3^	3.44 × 10^3^
*Q*_d1_ (s^n^_d1_ Ω^−1^ cm^−2^)	2.83 × 10^−5^	2.47 × 10^−5^
*n*_d1_	0.91	0.94
*R*_ct_ (Ω/cm^2^)	9.91 × 10^5^	3.99 × 10^5^
Χ^2^ (chi-squared)	1.9 × 10^−4^	1.5 × 10^−4^

**Table 4:** Jet electrochemical micromilling parameters.

Machining Condition	Parameter
Applied voltage (V)	35
Scanning speed (μm/s)	75
Machining gap (μm)	200
Electrolyte pressure (MPa)	1

## Data Availability

Data are contained within the article.
